# Prevalence and genotype analysis of *Cryptosporidium* spp*.* in nine species of wild rodents in China

**DOI:** 10.1051/parasite/2025012

**Published:** 2025-03-07

**Authors:** Zhen-Qiu Gao, Hai-Tao Wang, Jing-Hao Li, Yi-Xuan Song, Qing-Yu Hou, Si-Yuan Qin, He Ma, Quan Zhao, Ya Qin

**Affiliations:** 1 School of Pharmacy, Yancheng Teachers University Yancheng Jiangsu Province 224000 PR China; 2 College of Life Sciences, Changchun Sci-Tech University Shuangyang Jilin Province 130600 PR China; 3 College of Veterinary Medicine, Qingdao Agricultural University Qingdao Shandong Province 266109 PR China; 4 Center of Prevention and Control Biological Disaster, State Forestry and Grassland Administration Shenyang Liaoning Province 110034 PR China; 5 Forestry Investigation and Planning Institute of Liaoning Shenyang Liaoning Province 110000 PR China; 6 College of Veterinary Medicine, College of Animal Science and Technology, Jilin Agricultural University Changchun Jilin Province 130118 PR China

**Keywords:** *Cryptosporidium*, Genotypes, Prevalence, *SSU* rRNA gene, Wild Rodents, Zoonosis

## Abstract

*Cryptosporidium* is a significant zoonotic parasite with broad distribution in both humans and rodents. In this study, 510 fecal samples were collected from nine species of wild rodents across Guangxi, Yunnan, and Hunan Provinces in China. Nested PCR analysis targeting the *SSU* rRNA gene revealed an overall *Cryptosporidium* infection rate of 1.8% (9/510) among rodents in these provinces. The highest positivity rate was observed in Guangxi Province at 4.9% (5/103), followed by Yunnan Province (2.3%, 2/88), and Hunan Province (0.6%, 2/319). Notably, *Rattus losea* exhibited the highest prevalence rate at 9.8% (4/41), while *Rattus flavipectus* and *Niviventer lotipes* showed rates of 5.1% (2/39) and 4.4% (1/23), respectively. Various genotypes/species were identified, including *Cryptosporidium viatorum*, *Cryptosporidium muris*, *Cryptosporidium* vole genotype VII, and *Cryptosporidium ratti*, rat genotypes II, and IV. The study also found that wild rodents inhabiting mountainous areas had a higher prevalence rate at 4.9% (5/103) compared to those residing in fields and lake beaches, where prevalence rates were 2.1% (2/95) and 0.6% (2/312), respectively. This study provides new insights into *Cryptosporidium* infection rates among wild rodents and identifies two zoonotic species, *C. viatorum* and *C. muris*. These findings underscore the potential risk posed by Chinese wild rodent populations in transmitting zoonotic *Cryptosporidium*, which could significantly impact public health. Therefore, effective control strategies are needed to prevent transmission between humans and rodents.

## Abstract (fr)

*Cryptosporidium* est un parasite zoonotique important avec une large distribution chez l’homme et les rongeurs. Dans cette étude, 510 échantillons fécaux ont été collectés de neuf espèces de rongeurs sauvages dans les provinces du Guangxi, du Yunnan et du Hunan en Chine. L’analyse par PCR imbriquée ciblant le gène ARNr SSU a révélé un taux global d’infection par *Cryptosporidium* de 1,8 % (9/510) parmi les rongeurs de ces provinces. Le taux de positivité le plus élevé a été observé dans la province du Guangxi avec 4,9 % (5/103), suivie de la province du Yunnan (2,3 %, 2/88) et de la province du Hunan (0,6 %, 2/319). Notamment, *Rattus losea* a présenté le taux de prévalence le plus élevé à 9,8 % (4/41), tandis que *Rattus flavipectus* et *Niviventer lotipes* ont affiché des taux de 5,1 % (2/39) et 4,4 % (1/23), respectivement. Divers génotypes/espèces ont été identifiés, notamment *Cryptosporidium viatorum*, *Cryptosporidium muris*, *Cryptosporidium* de campagnol génotype VII et *Cryptosporidium ratti*, génotypes de rats II et IV. L’étude a également révélé que les rongeurs sauvages habitant les zones montagneuses avaient un taux de prévalence plus élevé, de 4,9 % (5/103), par rapport à ceux résidant dans les champs et les bords de lacs, où les taux de prévalence étaient respectivement de 2,1 % (2/95) et de 0,6 % (2/312). Cette étude apporte de nouvelles perspectives sur les taux d’infection à *Cryptosporidium* chez les rongeurs sauvages et identifie deux espèces zoonotiques, *C. viatorum* et *C. muris*. Ces résultats soulignent le risque potentiel posé par les populations de rongeurs sauvages chinois dans la transmission des *Cryptosporidium* zoonotiques, ce qui pourrait avoir des conséquences importantes sur la santé publique. Par conséquent, des stratégies de contrôle efficaces sont nécessaires pour prévenir la transmission entre les humains et les rongeurs.

## Introduction

*Cryptosporidium* is a significant zoonotic pathogen capable of infecting a wide range of hosts, including humans and wildlife [18]. Infections in humans and animals typically occur through the ingestion of water or food contaminated with *Cryptosporidium* oocysts [17]. While *Cryptosporidium* infections are often asymptomatic, they can sometimes lead to diarrhea and dehydration [10]. To date, over 170 species and genotypes of *Cryptosporidium* have been identified across various animal species [11, 36, 38]. Among these, more than 26 species and 56 genotypes have been detected in wild rodents [11]. *Cryptosporidium muris* was first identified in rodents in 1907 and has since been widely reported [4, 5, 47, 48]. Other species, such as *C. parvum*, *C. ubiquitum*, rodent genotype II, *C. hominis*, *C. meleagridis*, *C. andersoni*, *C. viatorum*, *C. alticolis*, *C. microti*, and *C. apodemi*, have also occasionally been reported in rodents [9, 21]. Recently, *C. ditrichi* was found in *Apodemus* species [11]. Notably, several of these species, including *C. ditrichi* [3], *C. parvum*, *C. hominis*, *C. muris* [6], *C. meleagridis*, *C. viatorum*, *C. andersoni*, and *C. ubiquitum*, have also been described in humans [12, 41].

Wild rodents are widely distributed all over the world, and can transmit many pathogens to humans and other animals, such as *Helicobacter* spp. [53], tick-borne encephalitis virus [2], and *Toxoplasma gondii* [32]. Although several studies have explored the prevalence of *Cryptosporidium* in wild rodents across different regions [31], data on certain rodent species remain limited. This study investigated the prevalence and risk factors associated with *Cryptosporidium* in nine wild rodent species from Yunnan, Guangxi, and Hunan Provinces in China, while also identifying the specific genotypes and species involved.

## Materials and methods

### Ethics statement

This study complied with the Animal Ethics Guidelines of China and was approved by the Institutional Animal Care and Use Committee of Yancheng Teachers University. All wild rodents were handled according to international welfare standards and university protocols to ensure humane treatment.

### Collection of samples

From August 2023 to May 2024, a total of 510 samples were collected from three provinces: Yunnan (*n =* 88), Hunan (*n* = 319), and Guangxi (*n* = 103). The species composition included *Microtus fortis* (*n* = 287), *Niviventer lotipes* (*n* = 23), *Rattus norvegicus* (*n* = 41), *Apodemus agrarius* (*n* = 22), *Rattus flavipectus* (*n* = 39), *Bandicota indica*, (*n =* 39), *Rattus rattus sladeni* (*n =* 5), *Rattus losea* (*n* = 41), and *Mus musculus* (*n* = 13). Rodents were trapped at 6-meter intervals, with melon seeds used as bait. Fresh fecal samples were then collected directly from the rectum of each captured rodent. During the sampling process, detailed information was recorded, including the species, gender, collection time, location, and specific habitat type, such as field, lake beaches, or mountainous area. The collected samples were promptly transported to the laboratory under controlled conditions with ice and stored at −20 °C for subsequent analysis.

### DNA extraction and PCR amplification

DNA was extracted from stool samples using an EZNA^®^ Stool DNA Kit (OMEGA Biotek Inc., Norcross, GA, USA) and stored at −20 °C until PCR amplification. The *SSU* rRNA gene of *Cryptosporidium* was amplified using nested PCR, with primers consistent with those used in previous studies [37]. The primer sequences were as follows: external primers F1 (5′-GACATATCATTCAAGTTTCTGACC-3′) and R1 (5′-CTGAAGGAGTAAGGAACAACC-3′), and internal primers F2 (5′-CCTATCAGCTTTAGACGGTAGG-3′) and R2 (5′-TCTAAGAATTTCACCTCTGACTG-3′). The PCR cycling conditions were as follows: 5 min at 95 °C, followed by 35 cycles of 30 s at 94 °C, 45 s at 58 °C, and 1 min at 72 °C, with a final extension at 72 °C for 10 min. All secondary PCR products were electrophoresed on 1% agarose gels containing Biosharp and visualized under UV light.

### Sequencing and phylogenetic analyses

All PCR products showing positive bands were sent to General Biology Co., LTD (Anhui, China) for sequencing. *Cryptosporidium* genotypes were determined by aligning the sequences with reference sequences in GenBank (http://www.ncbi.nlm.nih.gov/BLAST/). Phylogenetic trees were constructed using the neighbor-joining (NJ) method in MEGA 11 (Version 11.0.13), with bootstrapping performed using 1,000 replicates to assess the genetic relationships among *Cryptosporidium* species/genotypes.

### Statistical analysis

Univariate analysis was conducted using SPSS software (IBM Corp., Armonk, NY, USA), with Fisher’s exact test employed to assess the association between variables. Statistical significance was defined as *p* < 0.05, and effect sizes were expressed as odds ratios (OR) with corresponding 95% confidence intervals (95% CI).

Multivariable regression analysis was performed using SAS (SAS Institute Inc., Cary, NC, USA, Version 9.4) to assess the potential associations between *Cryptosporidium* infection and five independent variables: region (x1), species (x2), gender (x3), environment (x4), and season (x5). Each variable was included as an independent variable in the binary Logit model for multivariate regression analysis. The best model was selected based on Fisher’s scoring algorithm. All tests were two-sided, and results were considered statistically significant if *p* < 0.05 [52].

## Results

### Prevalence of *Cryptosporidium*

**Figure 1:**
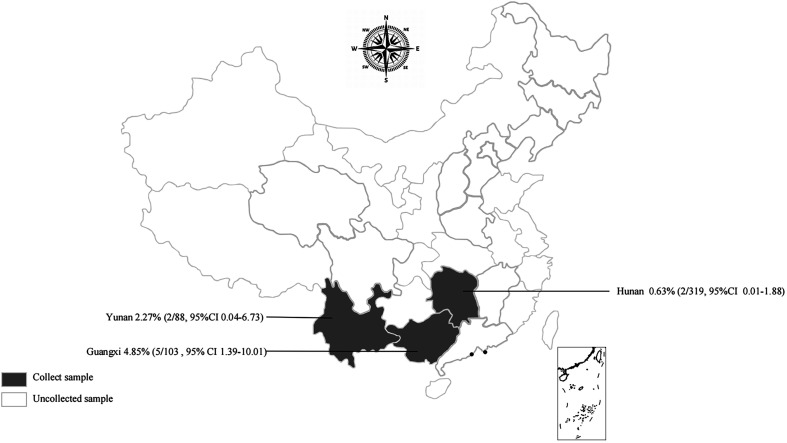
Map of the prevalence of *Cryptosporidium* in wild rodents in China.

Among the 510 samples analyzed, the overall prevalence of *Cryptosporidium* was 1.8% (9/510, 95% CI 0.81–3.32). The prevalence varied across the three provinces: 2.3% in Yunnan (2/88, 95% CI 0.04–6.73), 4.85% in Guangxi (5/103, 95% CI 1.39–10.01), and 0.6% in Hunan (2/319, 95% CI 0.01–1.88) (Fig. 1, Table 1). Among rodent species, *R. losea* exhibited the highest prevalence at 9.8% (4/41, 95% CI 2.20–21.07), followed by *R. flavipectus* at 5.1% (2/39, 95% CI 0.09–14.84), *N. lotipes* at 4.4% (1/23, 95% CI 0.00–17.73), and *Microtus fortis* at 0.7% (2/287, 95% CI 0.01–2.09). The prevalence rates between male and female rodents were 1.8% (5/272, 95% CI 0.52–3.84) and 1.7% (4/238, 95% CI 0.36–3.79), respectively. Rodents in mountainous areas had the highest infection rate at 4.9% (5/103, 95% CI 1.39–10.01), followed by field rodents at 2.1% (2/95, 95% CI 0.03–6.24) and those from lake beach habitats at 0.6% (2/312, 95% CI 0.01–1.92) (Table 1).

**Table 1:** Factors associated with the prevalence of *Cryptosporidium* in wild rodents in China.

Factors	Category	No. tested	No. positive	% (95% CI*)	*p*-value	OR (95% CI)
Region	Hunan Province	319	2	0.6 (0.01–1.88)	0.017	Reference
	Yunnan Province	88	2	2.3 (0.04–6.73)		3.63 (0.50–26.10)
	Guangxi Province	103	5	4.9 (1.39–10.01)		7.74 (1.48–40.51)
Species	*Microtus fortis*	287	2	0.7 (0.01–2.09)	0.004	Reference
	*Niviventer lotipes*	23	1	4.4 (0.00–17.73)		6.24 (0.55–71.42)
	*Rattus norvegicus*	41	0	0.00 (–)		–
	*Apodemus agrarius*	22	0	0.00 (–)		–
	*Rattus flavipectus*	39	2	5.1 (0.09–14.84)		7.36 (1.01–53.74)
	*Bandicota indica*	39	0	0.00 (–)		–
	*Rattus rattus sladeni*	5	0	0.00 (–)		–
	*Rattus losea*	41	4	9.8 (2.20–21.07)		14.00 (2.49–78.86)
	*Mus musculus*	13	0	0.00 (–)		–
Season	Summer	319	2	0.6 (0.01–1.88)	0.017	Reference
	Autumn	88	2	2.3 (0.04–6.73)		3.63 (0.503–26.101)
	Winter	103	5	4.9 (1.39–10.01)		7.74 (1.48–40.51)
Gender	Female	238	4	1.7 (0.36–3.79)	1.000	Reference
	Male	272	5	1.8 (0.52–3.84)		1.09 (0.29–4.12)
Environments	Lake beach	312	2	0.6 (0.01–1.92)	0.018	Reference
	Field	95	2	2.1 (0.03–6.24)		3.28 (0.47–23.63)
	Mountain	103	5	4.9 (1.39–10.01)		7.57 (1.45–39.62)
Total		510	9	1.8 (0.81–3.32)		

* CI: confidence interval.

### Association between positivity and exposure

Table 1 outlines the association between *Cryptosporidium* positivity and exposure variables such as age and region based on univariate analysis. Results of multivariate analysis showed that species emerged as the only significant factor retained in the final model, indicating its strong influence on *Cryptosporidium* infection. In this model, species significantly impacted infection risk, with *N. lotipes* (OR 6.24, 95% CI 0.55–71.42), *R. flavipectus* (OR 7.36, 95% CI 1.01–53.74), and *R. losea* (OR 14.00, 95% CI 2.49–78.86) showing greater susceptibility to *Cryptosporidium* compared to *Microtus fortis*.

### *Cryptosporidium* species/genotypes

**Figure 2:**
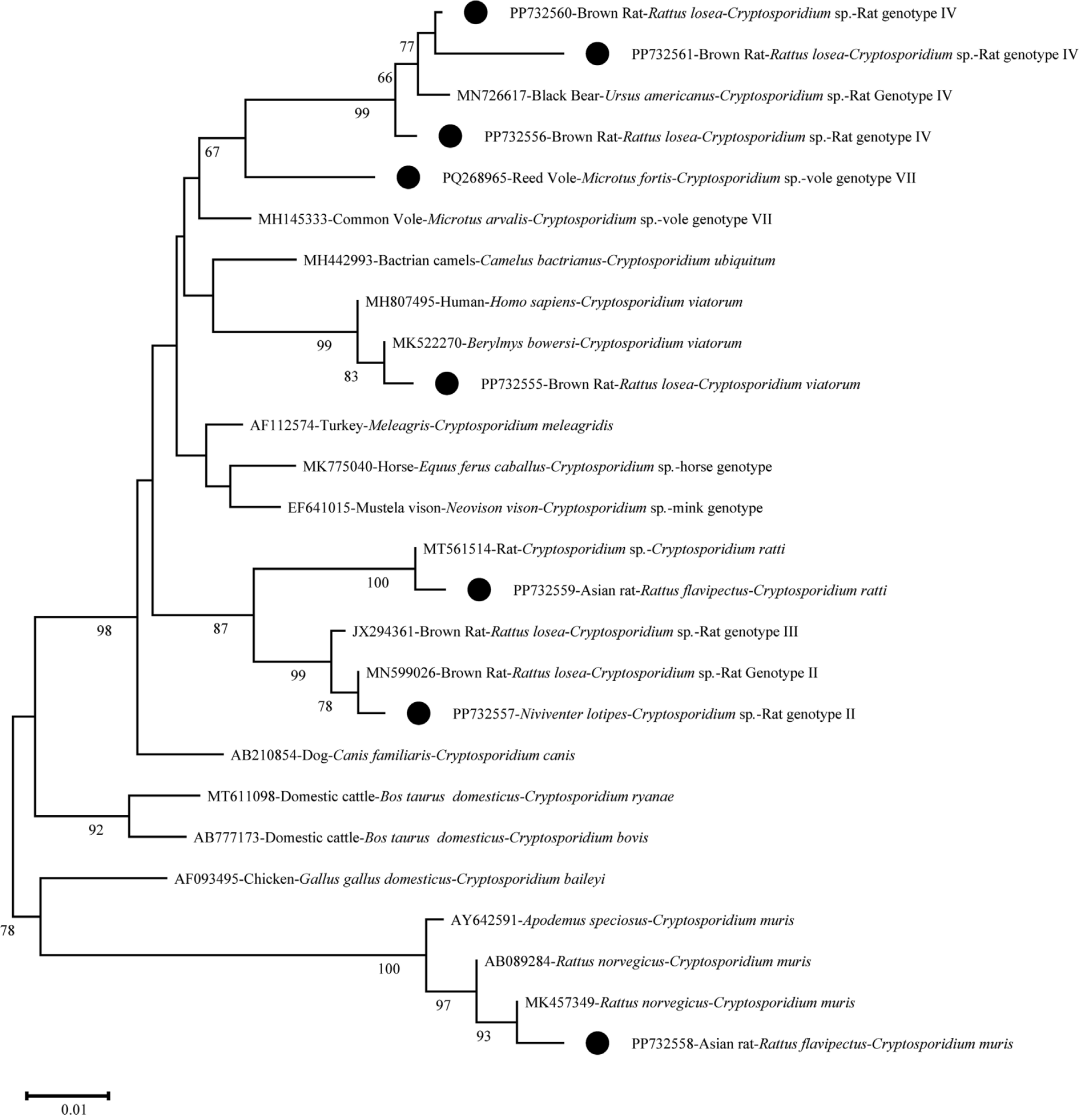
Phylogenetic relationships among *Cryptosporidium* spp. based on a neighbor-joining tree of the *SSU* rRNA gene. Cluster reliability was assessed through bootstrap analysis with 1,000 replicates; values above 50% are not shown. Black circles mark the *Cryptosporidium* species/genotypes identified in this study.

This survey identified six species/genotypes of *Cryptosporidium* in wild rodents: *C. viatorum*, *C. muris*, *Cryptosporidium* vole genotype VII, *C. ratti*, rat genotype II, and rat genotype IV. The zoonotic species *C. viatorum* and *C. muris* were found in rodents from field and mountainous regions, respectively (Table 2). The nucleotide sequences of the *Cryptosporidium SSU* rRNA gene obtained in this study matched those of *C. viatorum*, *C. muris*, *Cryptosporidium* vole genotype VII, and *C. ratti*, rat genotypes II, and IV in GenBank. Phylogenetic analysis using the NJ method clustered these sequences with the corresponding species/genotypes (Fig. 2).

**Table 2:** Distribution of *Cryptosporidium* species/genotypes.

Factors	Category	No. positive/tested (%)	Species/genotypes (No.)
Region	Hunan Province	2/319 (0.6)	*Cryptosporidium* vole genotype VII (*n* = 2)
	Yunnan Province	2/88 (2.3)	*C. muris* (*n* = 1), *C. ratti* (*n* = 1)
	Guangxi Province	5/103 (4.9)	*C. viatorum* (*n* = 1), Rat genotype II (*n* = 1), Rat genotype IV (*n* = 3)
Species	*Microtus fortis*	2/287 (0.7)	*Cryptosporidium* vole genotype VII (*n* = 2)
	*Niviventer lotipes*	1/23 (4.4)	Rat genotype II (*n* = 1)
	*Rattus norvegicus*	0/41 (0)	–
	*Apodemus agrarius*	0/22 (0)	–
	*Rattus flavipectus*	2/39 (5.1)	*C. muris* (*n* = 1), *C. ratti* (*n* = 1)
	*Bandicota indica*	0/39 (0)	–
	*Rattus rattus sladeni*	0/5 (0)	–
	*Rattus losea*	4/41 (9.8)	*C. viatorum* (*n* = 1), Rat genotype IV (*n* = 3)
	*Mus musculus*	0/13 (0.0)	–
Season	Autumn	2/88 (2.3)	*C. muris* (*n* = 1), *C. ratti* (*n* = 1)
	Summer	2/319 (0.6)	*Cryptosporidium* vole genotype VII (*n* = 2)
	Winter	5/103 (4.9)	*C. viatorum* (*n* = 1), Rat genotype II (*n* = 1), Rat genotype IV (*n* = 3)
Gender	Male	5/272 (1.8)	*C. viatorum* (*n* = 1), Rat genotype IV (*n* = 3), *Cryptosporidium* vole genotype VII (*n* = 1)
	Female	4/238 (1.7)	*C. muris* (*n* = 1), *C. ratti* (*n* = 1), Rat genotype II (*n* = 1), *Cryptosporidium* vole genotype VII (*n* = 1)
Environments	Field	2/95 (2.1)	*C. muris* (*n* = 1), *C. ratti* (*n* = 1)
	Lake beach	2/312 (0.6)	*Cryptosporidium* vole genotype VII (*n* = 2)
	Mountain	5/103 (4.9)	*C. viatorum* (*n* = 1), Rat genotype II (*n* = 1), Rat genotype IV (*n* = 3)
Total		9/510 (1.8)	*C. viatorum* (*n* = 1), *C. muris*(*n* = 1), *C. ratti* (*n* = 1), Rat genotype II (*n* = 1), Rat genotype IV (*n* = 3), *Cryptosporidium* vole genotype VII (*n* = 2)

## Discussion

Rodents are associated with over 60 zoonotic infectious diseases, posing a significant public health threat [29, 33]. *Cryptosporidium* are commonly found in wild rodents; furthermore, rodents can serve as carriers of *Cryptosporidium* to humans and livestock [39]. This study aimed to investigate the prevalence and genotype distribution of *Cryptosporidium* in wild rodents across three provinces in China, to improve our understanding of its occurrence in these animals.

The study revealed a prevalence of *Cryptosporidium* in wild rodents of 1.8%. A previous study from China reported a similar prevalence of 2.2% (11/498) [34]. In Yunnan Province, the prevalence was 2.3%, marking the first survey of *Cryptosporidium* in wild rodents in this region. In Guangxi Province, the prevalence was 4.9%, lower than the 9.5% reported in China (7/74) [34]. The prevalence observed in this study is significantly lower than the global average of 19.8% (4,589/23,142) [51]. Other countries have reported higher prevalence rates, such as France with 15.4% (18/117) [14], Spain with 12.3% (22/179) [15], and Australia with 7.6% (19/250) [13]. In North America and Europe, the combined prevalence was 33.2% (362/1,089) [42]. However, the prevalence in Portugal was 1.0% (3/290), which is lower than that observed in this study [31]. Factors such as rodent species, sampling time, and detection methods may influence the prevalence of *Cryptosporidium*, but the key factors remain unknown and require further investigation [49].

*Cryptosporidium* was detected in three of the nine wild rodent species examined, with the highest prevalence observed in *R. losea* (9.8%, 4/41), followed by *R. flavipectus* (5.1%, 2/39), *N. lotipes* (4.4%, 1/23), and *Microtus fortis* (0.7%, 2/287), indicating statistically significant differences. A study in Zhejiang Province, China, found a similarly high infection rate of *Cryptosporidium* in *R. losea* (11.1%, 2/18) [24]. Different rodent species exhibit varying rates of *Cryptosporidium* infection in China, with rates ranging from 1.4% to 100% in pet rodents, 4.0% to 73.9% in wild rodents, 2.1% to 29.5% in field rodents, and 0.6% to 8.6% in laboratory rodents [24, 34, 51]. Although the risk of *Cryptosporidium* infection and transmission in rodents is significant, no strong correlation has been established between infection rates and specific rodent species.

Seasonal variations were observed, with *Cryptosporidium* infections in rodents significantly higher during winter compared to summer and autumn (*p* < 0.05). Previous studies have also reported higher infection rates in rodents during winter [22]. However, some evidence suggests that infection rates gradually increase from spring to summer, peaking in autumn [25]. Additionally, prevalence rates varied across different environments, with the highest incidence in mountainous areas (4.9%), followed by field areas (2.1%), and no detections at lake beaches. The higher prevalence in mountainous areas may be due to the presence of grazing domestic animals, which facilitate mutual transmission between domestic animals and wild rodents [20, 29, 33]. Thus, caution should be exercised when selecting grazing areas to avoid locations frequented by wild rodents.

Over 26 species and 59 genotypes of *Cryptosporidium* have been reported in rodents [11]. In this study, six *Cryptosporidium* species/genotypes were identified, including *C. muris*, *C. viatorum*, *Cryptosporidium* vole genotype VII, and *C. ratti*, rat genotypes II, and IV. *Cryptosporidium muris* and *C. ratti* were detected in Yunnan Province, *C. viatorum* and rat genotypes II and IV were found in Guangxi Province, and *Cryptosporidium* vole genotype VII was identified in Hunan Province. *Cryptosporidium viatorum* and Rat genotype II had previously been reported in rodents from Guangxi [34]. While the pathogenicity of *C. ratti*, rat genotypes II and IV in humans or animals remains unclear, these genotypes have been documented in multiple countries, and their host range is expanding as research progresses [7, 19, 23, 27]. *Cryptosporidium* vole genotype VII was first detected in wild-caught common voles in 2018 [21]. Fortunately, *Cryptosporidium* vole genotype VII and *C. ratti*, rat genotypes II and IV have not been reported in human samples to date, suggesting limited public health significance, though further research is needed to confirm this.

The study identified two significant zoonotic *Cryptosporidium* species: *C. viatorum* and *C. muris*. *Cryptosporidium viatorum* has only been detected in humans, wild rodents, and municipal wastewater, and its origin – whether from humans or rodents – remains uncertain [26, 8, 55]. The detection of *C. viatorum* in wild rodents in Guangxi is particularly notable, given its prior identification in humans in Guangxi in 2020, suggesting a high likelihood of transmission between humans and wild rodents in the region [50]. In China, *C. viatorum* has also been reported in wild rodents from Hainan and Liaoning Provinces, underscoring the potential transmission risk posed by these rodents [55, 30]. Globally, *C. viatorum* has been identified in countries such as Australia [26], France [14], India [40], Ethiopia [46], and Denmark [44], indicating its widespread distribution.

gp60 is considered a key genetic marker for *C. viatorum* and *Cryptosporidium parvum,* and multiple studies have used the gp60 gene to subtype *C. viatorum* and *C. parvum* carried by wild rodents [18, 24, 43]. For example, among 25 wild rodent *C. viatorum*-positive samples, 17 were successfully subtyped, revealing their classification into the XVa and XVc subtype families [8]. However, in this study, only one *C. viatorum* positive sample was detected, and attempts to further subtype it using gp60 were unsuccessful. This issue may be attributed to the low concentration of DNA templates or the limited number of positive samples. Therefore, future studies should aim to expand the sample size to enhance the success rate of genotyping and to provide more comprehensive insights into the genetic diversity of *C. viatorum* in wild rodents.

*Cryptosporidium muris* shows a similar distribution pattern to *C. viatorum* in China, with no reported human infections in the country [55, 34, 30]. However, human infections by *C. muris* have been reported in countries such as Peru [45], Kenya [16], Slovakia [35], and Nigeria [1]. *Cryptosporidium muris* exhibits a broad host range, encompassing cats, dogs, and non-human primates [19]. Its prevalence is generally higher in *Mus musculus*, *Rattus rattus*, and *R. norvegicus* among wild rodents [28, 54]. This study represents the first detection of *C. muris* in *R. flavipectus* in Yunnan, expanding its known host range. The detection of *C. viatorum* in *R. losea* and *C. muris* in *R. flavipectus* suggests these rodent species may play a role in the transmission dynamics of zoonotic *Cryptosporidium*. Their widespread defecation in natural habitats significantly amplifies the risk of zoonotic *Cryptosporidium* transmission by wild rodents [33, 51]. Therefore, it is imperative that preventive measures be implemented from a “one health” perspective to disrupt both direct and indirect routes of zoonotic disease transmission mediated by wild rodents [51].

## Conclusion

This study highlights the prevalence of *Cryptosporidium* in wild rodents across China, identifying six genotypes/species, including two zoonotic species, *C. viatorum* and *C. muris*. Notably, this is the first identification of the zoonotic *C. muris* in *R. flavipectus* in Yunnan Province. Additionally, the study showed a genetic link between *C. viatorum* found in wild rodents in Guangxi and in humans, underscoring the role of wild rodents as reservoirs of *Cryptosporidium* with potential transmission to humans and domestic animals. Therefore, continuous monitoring of *Cryptosporidium* prevalence in wild rodents is essential.

## Data Availability

The gene sequences obtained in the present study have been submitted to GenBank (accession nos. PP732555–PP732561, PQ268965).
